# Size of Patent Ductus Arteriosus and Echocardiographic Markers of Shunt Volume in Preterm Infants Based on Postnatal Age

**DOI:** 10.3389/fped.2021.635616

**Published:** 2021-04-20

**Authors:** Eui Kyung Choi, Kyu Hee Park, Byung Min Choi

**Affiliations:** Department of Pediatrics, Korea University College of Medicine, Seoul, South Korea

**Keywords:** preterm infant, hemodynamically significant patent ductus arteriosus, patent ductus arteriosis, early targeted treatment, echocardiography

## Abstract

**Objective:** This study was conducted in order to compare the strength of correlation between echocardiographic markers of shunt volume and patent ductus arteriosus (PDA) diameter based on postnatal age.

**Methods:** This retrospective study focused on preterm infants (aged <32 weeks of gestation) admitted to the Neonatal Intensive Care Unit of Korea University Ansan Hospital, between April 2014 and December 2017, who studied serial targeted neonatal echocardiography (TNE) for PDA during hospitalization. The association between echocardiographic characteristics and duct size was divided into the following days: within 3 days (very early, VE), 4–7 days after birth (early, E), and after 8 days of birth (late, L).

**Results:** We found 113 assessments conducted on 57 infants in the VE period, 92 assessments on 40 infants in the E period, and 342 assessments on 37 infants in the L period. Median gestational age and birth weight were 28^+2^ weeks of gestation and 1,115 g, respectively. In the univariate regression analysis, we found a statistically significant correlation between PDA diameter and all TNE markers in the E and L days, but not in the VE period. Only ductal velocity [coefficient of determination (*R*^2^) = 0.224], antegrade left pulmonary artery diastolic flow velocity (*R*^2^ = 0.165), left ventricular output (LVO)/superior vena cava (SVC) flow ratio (*R*^2^ = 0.048), and E/A wave ratio (*R*^2^ = 0.092) showed weak correlations with PDA diameter in the VE period. The slopes of the regressions showed significant changes based on postnatal age in the maximum ductal velocity, left atrium/aorta ratio, LVO/SVC flow ratio, and LVO.

**Conclusions:** It is difficult to predict the echocardiographic markers of shunt volume based on the PDA diameter in preterm infants younger than 4 days. A better understanding of the changes in the hemodynamic consequences of PDA based on postnatal age is needed when considering treatment.

## Introduction

Echocardiography is currently the preferred tool to screen neonates with hemodynamically significant patent ductus arteriosus (hsPDA) in the early postnatal period, owing to its potential benefits in the prevention of pulmonary hemorrhage or intra ventricular hemorrhage (1). The investigation is indicated for clinically suspected PDA, especially in very low birth weight neonates during the initial 24 to 72 h immediately following birth (2–4).

This transitional period is more complicated in preterm infants who have immature organ systems, thereby necessitating surfactant administration, ventilatory support, and vasoactive medications. The preterm left ventricular myocardium is exposed to an abrupt increase in afterload while being relatively immature; therefore, it may be less tolerant to the concurrent changes in preload conditions caused by the presence of pathologic atrial and ductal shunts. Therefore, defining hsPDA and deciding on the treatment may be complicated in the early postnatal period (5–7).

Since hsPDA is determined by both ductal size and shunt volume markers of pulmonary over circulation and systemic hypoperfusion in targeted neonatal echocardiography (TNE), the most common definition of hsPDA is based on the transductal diameter (2, 8, 9). Recently, Fernando de Freitas Martins et al. evaluated the correlation between ductal diameter and individual echocardiographic markers of shunt volume in preterm infants older than 7 days (10). However, there is lack of information regarding differences in the association between PDA diameter and shunt volume markers in the early postnatal period.

The aim of the present study was to compare the strength of association between echocardiographic markers of shunt volume and PDA diameter based on postnatal age and categorized as very early (VE, within 3 days after birth), early (E, 4–7 days after birth), and late (L, >8 days after birth) days.

## Materials and Methods

We retrospectively reviewed the medical records of 128 preterm infants who were born at <32 weeks of gestation and were admitted to the neonatal intensive care unit of Korea University Ansan Hospital between April 2014 and December 2017. Infants with major congenital anomalies (except PDA and patent foramen ovale), chromosomal abnormalities, and incomplete medical records were excluded. An additional exclusion criterion was the absence of data on TNE assessment of hemodynamic significance of the PDA during hospitalization.

The study was approved by the Institutional Review Board of the Korea University Ansan Hospital (2019AS0223).

### Echocardiography Assessment

All infants delivered before 32 weeks of gestation underwent echocardiographic screening to identify congenital heart anomalies. After screening, we decided to study serial TNE assessments when the infants had PDA-related clinical signs or an established shunt, defined as evidence of left-to-right dominant transductal flow to ensure sufficient shunt flow. We performed standardized TNE assessment of hemodynamic significance of the PDA with pulmonary over circulation, myocardial performance, and/or systemic hypoperfusion to decide on the course of treatment. Given the direct vaso constricting effect of ibuprofen, TNE assessments were always performed before each dose of the medication. All study measurements were recorded by a single operator using the Vivid q system (GE Healthcare, Haifa, Israel) and a 10-Hz probe. Analysis of the recorded echocardiographic markers was performed by a blinded investigator. The mean variability between measurements was within 20% for all parameters, and 90% of the measures were approximately within 15% of each other.

Two-dimensional, M-mode, pulse, and color flow Doppler imaging were performed. PDA diameter was measured at its narrowest point, usually at the pulmonary end. We measured additional parameters, including ductal velocity (Vmax, peak velocity of transductal flow was assessed by pulse- and continuous-wave Doppler), end diastolic velocity of the left pulmonary artery (LPA), ratio of the left atrial diameter to aortic root diameter (LA/Ao, measured from the long axis parasternal view at the level of the aortic valve, using M-mode), ratio of mitral inflow E wave peak velocity to A wave peak velocity (E/A wave ratio, trans-mitral valve flow was assessed by pulse-wave Doppler at the tips of the mitral valve leaflets from an apical four-chamber view), left ventricular output (LVO, calculated based on heart rate, aortic diameter, and velocity time integral), and the percentage of retrograde diastolic flow in the descending aorta distal to PDA.

### Clinical Data

The medical records of all eligible infants were reviewed to obtain neonatal demographic and clinical data. Birth and clinical characteristics were collected, including gestational age (GA) and birth weight at delivery, sex, mode of delivery, 5-min Apgar score, the use of antenatal steroids, and premature membrane rupture. The following clinical outcomes were also recorded: surfactant use in respiratory distress syndrome, PDA treatment with intravenous ibuprofen or surgical ligation, necrotizing enter colitis with radiologic evidence of pneumatosis intestinalis, intra ventricular hemorrhage classified according to the Papile classification, bronchopulmonary dysplasia (defined as the requirement for supplemental oxygen with a fraction of inspired oxygen of more than 0.21 for 28 days of life), treated retinopathy of prematurity, and death before discharge.

The policies of management to PDA in our NICU were “symptomatic treatment” and/or “early targeted treatment.” The cyclooxygenase inhibitor was administered intravenously after confirming the diagnosis of hsPDA. Early targeted treatment based on echocardiographic parameters may allow for the selection of high-risk preterm infants prior to the duct becoming clinically significant. The clinical signs of PDA were based on the following features: (1) the presence of a systolic or continuous murmur; (2) a bounding pulse or a hyperactive precordial pulse; (3) difficulty in maintaining the blood pressure, i.e., hypotension (the lower limit of normal mean arterial pressure was regarded to corrected gestational age) without response to loading fluid and infusion of dopamine; (4) a worsening ventilator status; and (5) chest radiographic evidence, i.e., pulmonary congestion or cardiomegaly (a cardiothoracic ratio >60%) with increased pulmonary flow. Symptomatic PDA was defined as the presence of two of these five signs with the confirmation of a large left to right ductal flow by color flow Doppler echocardiography (11).

### Statistical Analysis

Statistical analyses were performed using IBM SPSS 20 and R statistical software. Continuous variables were analyzed using either the *t* test or the Mann–Whitney *U* test/Kruskal–Wallis test for normal or skewed distributions. Proportions were tested using the chi-squared test and Fisher's exact test. *p* values of <0.05 were considered statistically significant. Data are presented as mean ± standard deviation (SD), median with interquartile range (IQR), or rate.

The association between PDA size and each of the echocardiographic markers was assessed using the Pearson correlation coefficient, followed by a linear regression analysis. The association was divided into VE, E, and L days as explained previously. The coefficient of determination (*R*^2^) was used to determine the strength of the association for each marker. We used the *F* test and *t* test to compare the coefficients and slopes across different regressions.

## Results

**Figure 1:**
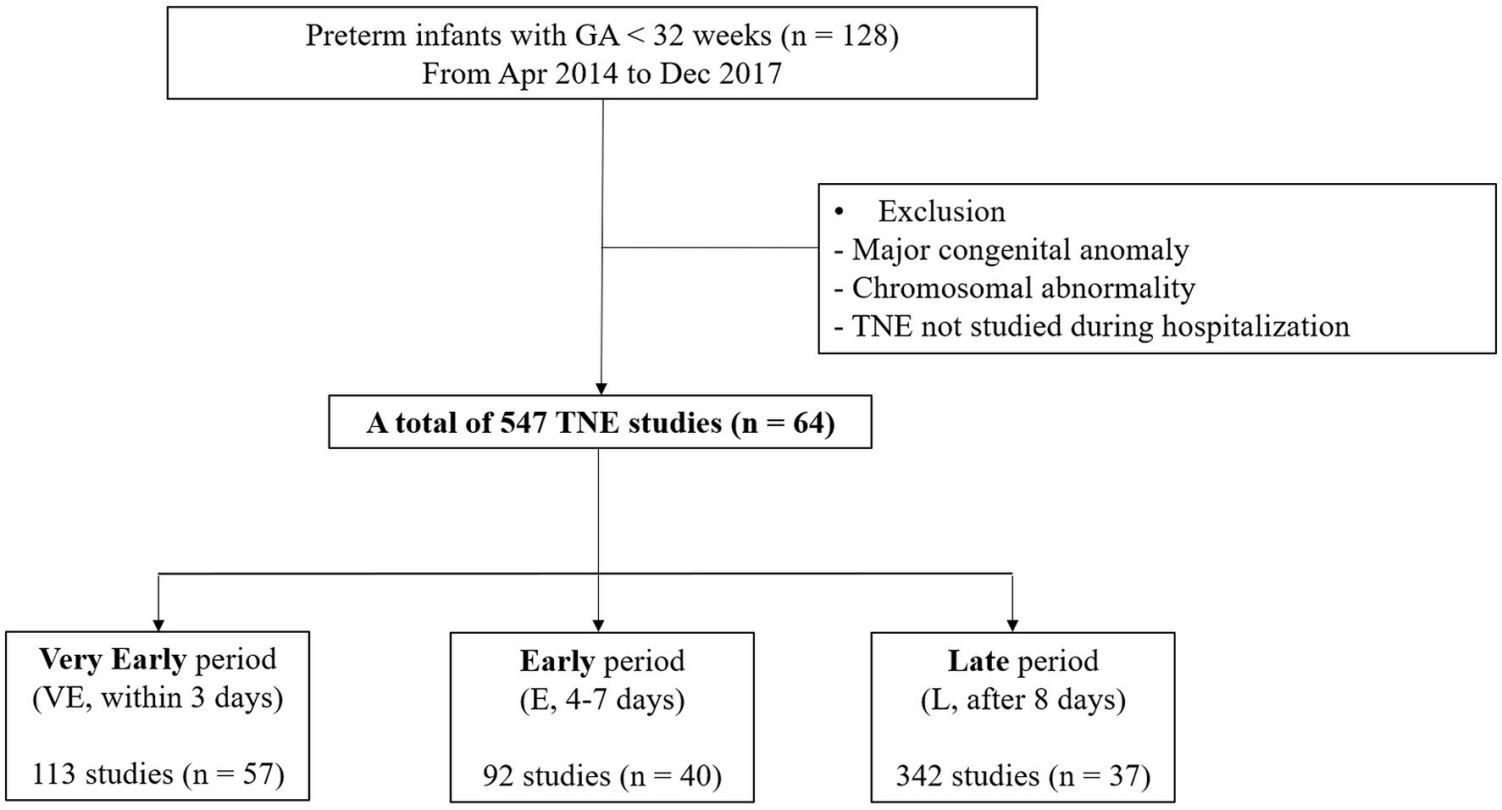
Flowchart of the study population.

A total of 128 preterm infants born at <32 weeks of gestation were identified, and 64 were excluded due to the presence of congenital defects, chromosomal abnormality, and absence of TNE data. A total of 547 TNE assessments in 64 infants with PDA were analyzed. We found 113 assessments conducted on 57 infants in the VE period, 92 assessments on 40 infants in the E period, and 342 assessments on 37 infants in the L period (Figure 1). Median GA and birth weight were 28^+2^ weeks (IQR, 26^+4^-29^+6^ weeks) and 1,115 g (IQR, 950–1,397 g) (Table 1). The median number of TNE assessments per infant was 6 (IQR, 3–13). These were performed at a median of 2 days (range, 0–3) after birth in the VE period, 5 days (range, 4–7) after birth in the E period, and 18 days (range, 8–63) after birth in the L period. No difference was observed in the GA and birth weight of infants among the three periods. There were significant differences in the TNE assessments between the groups according to postnatal age, except E/A wave ratio, and in the use of mechanical ventilator depending on the different postnatal days (77.1 vs. 47.8 vs. 35.9%, *p* < 0.05). There were no significant differences in receiving treatment among the three periods (Table 2).

**Table 1:** Demographics and outcomes.

**Characteristics and outcomes**	**Values**
Gestational age, weeks, median (IQR)	28^+2^ (26^+4^-29^+6^)
Birth weight, g, median (IQR)	1,115 (950–1,397)
Number of echocardiograms, median (IQR)	6 (3–13)
Male sex, *n* (%)	44 (68.8)
PPROM, *n* (%)	17 (26.6)
Antenatal steroids (complete), *n* (%)	36 (56.3)
Surfactant administration, *n* (%)	57 (89.1)
Bronchopulmonary dysplasia, *n* (%)	37 (57.8)
Necrotizing enterocolitis, *n* (%)	5 (7.8)
Intraventricular hemorrhage grades 3–4, *n* (%)	7 (10.9)
Retinopathy of prematurity (laser/bevacizumab), *n* (%)	1 (1.6)
Death, *n* (%)	5 (7.8)
PDA, *n* (%)	
No treatment	19 (29.7)
Pharmacological treatment	30 (46.9)
Pharmacological treatment and ligation	6 (9.3)
Ligation	2 (3.1)

**Table 2:** Clinical characteristics and echocardiographic markers of shunt volume based on postnatal age.

	**Very early (*n* = 113)**	**Early (*n* = 92)**	**Late (*n* = 342)**	***p***
Numbers of infants during the period, *n*	57	40	37	
Gestational age, weeks, median (IQR)	28^+2^ (26^+4^-29^+6^)	27^+6^ (26^+4^-29^+6^)	27^+1^ (26^+0^-30^+0^)	0.536
Birth weight, g, median (IQR)	1,115 (950–1,415)	1,095 (940–1,367)	1,040 (880–1,315)	0.491
Mechanical ventilation during assessments, *n* (%)	87/113 (77.1)	44/92 (47.8)	123/342 (35.9)	<0.001
Pharmacological treatment for PDA, *n* (%)	30/58 (53.6)	23/40 (59.0)	22/37 (61.1)	0.492
PDA diameter (cm)	1.20 (1.03–1.50)	1.06 (0.90–1.31)	1.28 (1.00–1.68)	0.000
Ductal velocity max (m/s)	1.55 (1.18–2.19)	2.06 (1.48–2.50)	2.28 (1.68–2.92)	0.000
Antegrade LPA diastolic flow (cm/s)	23.88 (18.54–31.05)	24.55 (20.28–29.63)	27.23 (23.50–33.43)	0.000
Left atrial:aortic ratio	1.24 (1.18–1.33)	1.30 (1.20–1.38)	1.40 (1.27–1.50)	0.000
LVO/SVC flow ratio	1.57 (1.29–2.03)	1.63 (1.33–1.98)	1.93 (1.55–2.32)	0.000
E wave/A wave ratio	0.81 (0.77–0.86)	0.80 (0.76–0.87)	0.80 (0.75–0.85)	0.227
IVRT (ms)	59.15 (51.76–66.54)	51.81 (48.06–59.15)	48.06 (44.36–53.60)	0.000
Retrograde diastolic flow (%)	0 (0–23.56)	0 (0–18.23)	0 (0–17.92)	0.000
Left ventricular output (ml/kg/min)	202.0 (169.5–240.0)	240.5 (192.8–293.0)	296.0 (236.8–376.3)	0.000

*Data are presented as median with interquartile range (IQR) for non-normally distributed variables. p values were obtained by Kruskal–Wallis test. PDA, patent ductus arteriosus; LPA, left pulmonary artery; LVO, left ventricular output; SVC, superior vena cava; IVRT, isovolumic relaxation time*.

**Figure 2:**
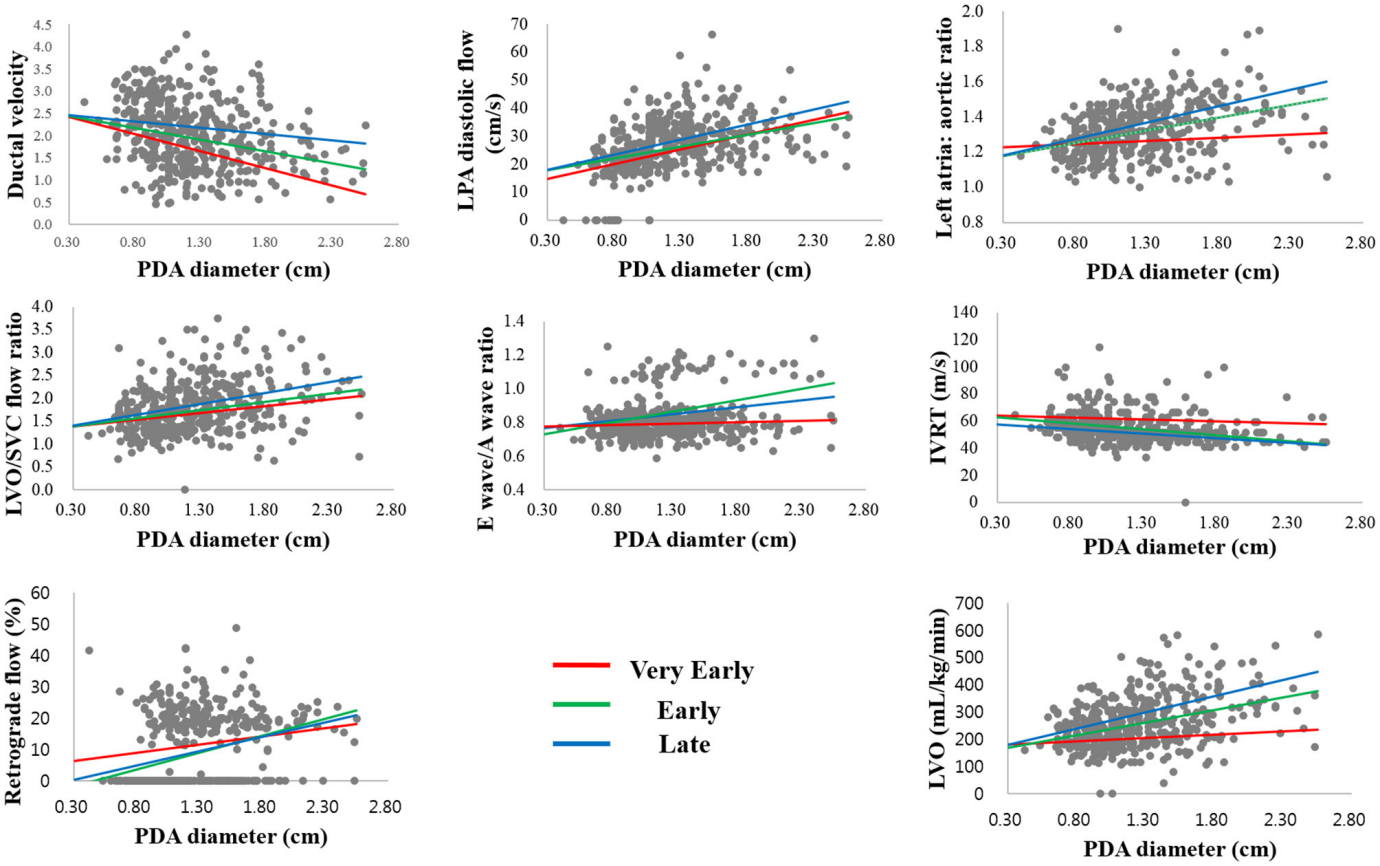
Scatterplots showing the association between the echocardiographic markers with PDA diameter in the three periods.

In the univariate regression analysis, we found a statistically significant correlation between PDA diameter and all TNE markers in the E and L days, but not in the VE period. Only ductal velocity (*R*^2^ = 0.224, *p* < 0.05), antegrade LPA diastolic flow (*R*^2^ = 0.165, *p* < 0.05), LVO/superior vena cava (SVC) flow ratio (*R*^2^ = 0.048, *p* < 0.05), and E/A wave ratio (*R*^2^ = 0.092, *p* < 0.05) showed weak correlations with PDA diameter in the VE period. The LA/Ao ratio (*R*^2^ = 0.010, *p* = 0.291) and LVO (*R*^2^ = 0.028, *p* = 0.078) did not correlate with PDA diameter in the VE period; however, there were strong correlations in the E (*R*^2^ = 0.230, *p* < 0.05 and *R*^2^ = 0.344, *p* < 0.05, respectively) and L days (*R*^2^ = 0.311, *p* < 0.05 and *R*^2^ = 0.272, *p* < 0.05, respectively) (Table 3). The regression lines in the scatter plots of PDA diameter and each of the TNE markers are outlined in Figure 2. *P-*values were analyzed to test the difference in the regression lines and slopes among groups. The regression lines of all echocardiographic markers except IVRT were significantly different between the VE and L days (Table 4). The slopes of the regressions showed significant changes depending on the time of birth for the maximum ductal velocity, LA/Ao ratio, LVO/SVC flow ratio, and LVO (Table 5). The slopes of the regressions for all markers except the maximum ductal velocity were statistically equivalent between the E and L days.

**Table 3:** Univariate regression analysis of PDA diameter and echocardiographic markers of shunt volume based on postnatal age.

**Markers**	**Very early**			**Early**			**Late**		
	***R*^**2**^**	**β coefficient correlation**	***p***	***R*^**2**^**	**β coefficient**	***p***	***R*^**2**^**	**β coefficient**	***p***
Ductal velocity Vmax (m/s)	0.224	−0.777	0.000	0.091	−0.529	0.003	0.000	0.024	0.823
Antegrade LPA diastolic flow (cm/s)	0.166	10.632	0.000	0.144	8.502	0.000	0.175	9.192	0.000
LA/Ao ratio	0.010	0.036	0.291	0.230	0.145	0.000	0.311	0.210	0.000
LVO/SVC flow ratio	0.048	0.293	0.020	0.083	0.360	0.006	0.183	0.584	0.000
E wave/A wave ratio	0.059	0.083	0.010	0.160	0.135	0.000	0.033	0.062	0.001
IVRT (ms)	0.009	−2.990	0.325	0.099	−8.976	0.002	0.155	−7.043	0.000
Retrograde diastolic flow (%)	0.021	5.272	0.122	0.167	10.800	0.000	0.087	7.073	0.000
LVO (ml/kg/min)	0.028	24.206	0.078	0.344	92.591	0.000	0.272	128.940	0.000

*LA/Ao, ratio of the left atrial diameter to aortic root diameter*.

**Table 4:** Comparing the linear regression lines by analysis of the covariance.

	**Very early vs. early**		**Early vs. late**		**Very early vs. late**	
	***t***	***p***	***t***	***p***	***t***	***p***
Ductal velocity Vmax (m/s)	4.61,728	0.011	8.832,969	0.000	39.4,595	0.000
Antegrade LPA diastolic flow (cm/s)	0.847,749	0.430	3.744,606	0.024	15.20,804	0.000
LA/Ao ratio	0.686,919	0.504	1.676,959	0.188	5.061,182	0.007
LVO/SVC flow ratio	6.722,909	0.002	8.450,433	0.000	48.53,806	0.000
E wave/A wave ratio	0.317094	0.729	5.835,583	0.003	13.93,361	0.000
IVRT (ms)	0.845,727	0.431	2.88,524	0.057	0.919,061	0.397
Retrograde diastolic flow (%)	8.801,438	0.000	9.717,368	0.000	72.00,497	0.000
LVO (ml/kg/min)	2.446,328	0.089	1.066,506	0.345	5.463,855	0.005

*p value < 0.05 means the lines are significantly different*.

**Table 5:** Comparing the slopes of linear regression lines by analysis of the covariance.

	**Very early vs. early**		**Early vs. late**		**Very early vs. late**	
	***t***	***p***	***t***	***p***	***T***	***p***
Ductal velocity Vmax (m/s)	−1.12842	0.260	−2.3712	0.018	−3.780218	0.000
Antegrade LPA diastolic flow (cm/s)	0.6711137	0.503	−0.2831184	0.777	0.6102204	0.542
LA/Ao ratio	−2.390946	0.018	−1.766935	0.078	−4.738054	0.000
LVO/SVC flow ratio	−0.3765719	0.707	−1.503919	0.133	−2.057582	0.040
E wave/A wave ratio	−1.131472	0.260	1.799364	0.073	0.5451293	0.586
IVRT (ms)	1.419482	0.157	−0.8386917	0.402	1.715798	0.0869
Retrograde diastolic flow (%)	−1.266117	0.207	1.324976	0.186	−0.6051878	0.545
LVO (ml/kg/min)	−3.544158	0.000	−1.51831	0.130	−4.679049	0.000

*p value < 0.05 means the slopes are significantly different*.

## Discussion

This study focused on the diameter of the PDA and its association with echocardiographic consequences of the shunt volume at the very early stages of life. It is difficult to estimate the echocardiographic markers of the ductal shunt and systemic hypoperfusion based on the PDA diameter in preterm infants younger than 4 days.

McNamara and Sehgal suggested a PDA staging system using clinical signs and echocardiographic parameters to understand the impact of the ductal shunt and PDA treatment (12, 13). However, their echocardiographic parameters have not been widely adopted in standard practice because of their extensive number of parameters and difficulties (14). Conventional echocardiographic markers such as PDA diameter do not predict neonatal outcomes, and it remains the routinely used parameter in clinical settings (15, 16). Based on the observations of our study, the PDA staging system was not correlated with the PDA diameter in the early period. We speculate that the impact of the PDA in echocardiography is less significant in the first 3 days of life due to the immediate transitional phase. The magnitude of the transductal shunt not only relates to the transductal diameter but also is affected by pulmonary and systemic vascular resistance and the compensatory ability of the immature myocardium depending on the time of birth (13). This result closely aligned with an earlier study reporting that clinical signs are not reliable in the first few days of life (17).

El-Khuffash et al. (18) proposed the use of another PDA severity score on day 2 after birth to predict the later occurrence of CLD/death. They suggested a predictive score within the first 48 h of life that could correlate with PDA-related morbidity, including GA, PDA diameter, LVO, maximum shunt velocity across PDA, and LV A wave. This suggestion is in accordance with the findings of our study, wherein the aforementioned factors showed a reliable correlation with the PDA diameter, despite being in the VE period. These factors could be predictive of the hemodynamic significance that can guide treatment decisions in the early period.

The treatment intervention for PDA in preterm infants varies from the conservative approach (“wait and watchful”) to the active, early treatment approach. Recent evidence suggests that mortality may depend on the management of PDA, and a moderate approach is associated with optimal outcomes (19). While in some infants a careful “wait and watchful” strategy may be the best option, other infants may need treatment to minimize the comorbidities in the early postnatal period (20). Also, for extremely preterm infants in the transitional period, echocardiographic assessment for hsPDA is very important for the conservative management of hemodynamically unstable infants. Our report is the first to directly investigate the strength of association between echocardiographic markers of shunt volume and PDA diameter based on postnatal age in preterm infants. Our results might help to select newborns in the early postnatal period that should be treated in order to improve their clinical outcomes without a significant increase of markers of pulmonary over circulation and/or systemic hypoperfusion. PDA symptoms and echocardiography results are insignificant in the first few days of life; therefore, comprehensive interpretation is essential before any intervention including conservative management, along with assessment of other factors such as GA at birth, clinical risk, ventilation requirements, cardiac biomarkers, and organ perfusion.

Our study is limited by its retrospective design and small sample size. The indication for TNE evaluation was at the discretion of the attending neonatologist, which may have introduced a selection bias toward sicker patients. Although we did not report the clinical effectiveness of PDA treatment at any of the included periods, the clinical effectiveness of treatments of PDA cannot be concluded because of the retrospective design. Of note, there were no significant differences in receiving treatment among the three periods. Since the rates of hsPDA vary widely by center, our results may not be generalizable to other centers where the rates of hsPDA differ from ours. There is an urgent need of prospective randomized controlled trials to assess the effectiveness of targeted treatment of hsPDA in matched conditions, including the timing, medications used, GA, and PDA severity in order to assess the association between PDA treatment and outcomes in preterm infants.

In conclusion, we found a significantly weaker association between PDA diameter and echocardiographic parameters of shunt volume in the VE days after birth than in the other periods. It is difficult to predict the echocardiographic markers of ductal shunt by the PDA diameter in infants younger than 4 days. A prospective study identifying the proper criteria for beneficial PDA treatment, especially in the early period after birth, is of paramount importance.

## Data Availability Statement

The original contributions presented in the study are included in the article/supplementary material, further inquiries can be directed to the corresponding authors.

## Ethics Statement

The studies involving human participants were reviewed and approved by Institutional Review Board of the Korea University Ansan Hospital. Written informed consent from the participants' legal guardian/next of kin was not required to participate in this study in accordance with the national legislation and the institutional requirements.

## Author Contributions

EC: primary author and responsible for data collection, data analysis, and manuscript writing. KP: data collection. BC: writing of the manuscript draft, editing, critical revision of the manuscript, and responsible for the overall content. All authors provided contributions and have read and approved the final version.

## Conflict of Interest

The authors declare that the research was conducted in the absence of any commercial or financial relationships that could be construed as a potential conflict of interest.
